# Coaching Leadership and structural empowerment of nurses in mobile and fixed Pre-Hospital Care

**DOI:** 10.1590/1980-220X-REEUSP-2024-0450en

**Published:** 2026-01-09

**Authors:** Letícia Santiago Utsch, Fernanda Oliveira Santos, Katharina Júlia Lacerda Oliveira, Hercules de Oliveira Carmo, André Almeida de Moura

**Affiliations:** 1 Universidade Federal de Minas Gerais, Escola de Enfermagem, Belo Horizonte, MG, Brazil.; 2 Universidade de São Paulo, Escola de Enfermagem, Departamento de Orientação Profissional, São Paulo, SP, Brazil.

**Keywords:** Leadership, Empowerment, Emergency Nursing, Nurses, Emergency Medical Services

## Abstract

**Objective:**

To analyze the correlation between Coaching Leadership exercised by mobile and fixed pre-hospital care (APH) nurses and the structural empowerment of these professionals.

**Method:**

Analytical and descriptive study with 79 mobile and fixed pre-hospital nurses. The following questionnaires were used: Characterization of subjects; Nurse Self-Perception Questionnaire in the Exercise of Leadership; and Work Effectiveness Conditions Questionnaire II (CET-II). Descriptive analysis and inferential analysis were used with Spearman’s test to determine the correlations between the dimensions of the instrument, adopting a significance level of 5%.

**Results:**

Coaching Leadership positively influences the empowerment of nurses working in the APH context, as evidenced by the weak correlation between the total scores of the instruments applied in the research (R = 0.376; p-value = <0.001). “Communication” correlated with all domains of CET-II, including the “total score” of the instrument.

**Conclusion:**

It is important that mobile and fixed APH organizations invest in the leadership of their nurses, based on the use of the Coaching Leadership model, especially considering that it has a positive influence on nurse empowerment.

## INTRODUCTION

The role of nurses in urgent and emergency care is of great importance, given that these professionals are in direct contact with patients from the very first moment (at the scene) through mobile Pre-Hospital Care (APH in the Portuguese acronym), in the form of the Mobile Emergency Care Service (SAMU in the Portuguese acronym), through fixed pre-hospital care, which includes Emergency Care Units (UPA in the Portuguese acronym), to the hospital that receives and provides intra-hospital care for acute health needs. In all these care scenarios, nurses are required to have attitudes, skills, and technical and scientific knowledge, in addition to observing ethical aspects related to urgency and emergency^(1)^.

In Brazil, the Urgency and Emergency Care Network (RUE in the Portuguese acronym) aims to develop actions for health promotion, prevention, and surveillance, as well as care and treatment for users in acute conditions. Against this background, the work process in urgent care requires significant dynamism on the part of professionals in response to high demands, unexpected events, constant professional training, synergy, and intercommunication among the team, both pre-hospital and intra-hospital, to promote safe and quality care^(2)^.

Thus, considering this dynamism, nurses play essential roles in this care, especially in the use of leadership in their professional practice. It is through this competence that such professionals achieve better results for patients and teams and promote dialogue with other health services and sectors^(3,4)^. Therefore, it is clear that the leadership of nurses in this context seeks to favor the dimensions of their work process^(5)^.

A recent editorial^(6)^ in an international emergency nursing journal reflects on leadership as a fundamental competency for nurses working in the emergency sector and states that, in order to effectively mobilize this competency, these professionals must constantly update their knowledge and combine leadership with the use of other competencies and management tools. Therefore, in order for nurses to exercise this competency, they must overcome certain challenges, such as work overload and high demand, using strategies to overcome them. To this end, the text points out that, in addition to leadership, nurses need to master a wide range of tools such as strategic thinking, problem solving, emotional intelligence, collaboration, team building, and decision making.

Corroborating this editorial, an ethnographic study with Swedish pre-hospital nurses states that, based on their responsibility and leadership, nurses have a key role in elucidating the values of their subordinates, encouraging them to develop their own qualities, thereby promoting a favorable practice environment in the nursing team^(7)^.

Given the magnitude of leadership for nurses working in emergency situations, the Coaching Leadership model is pointed out as a reference for the exercise of this competence by nurses. Conceptually, Coaching Leadership is based on the coaching process, which consists of a competence through which the leader seeks to influence the team to achieve objectives, while enabling the development of their subordinates. To this end, this model is based on four dimensions of the coaching process, namely: “Communication,” “Giving and receiving feedback,” “Empowering and influencing,” and “Supporting the team.”^(4,8)^

Based on this leadership model in APH, two Brazilian articles^(4,9)^, developed in the state of Goiás, revealed that nurses, when performing Coaching Leadership, promote positive changes in their workplace, such as the satisfaction of their subordinates and, likewise, enable the provision of quality and safe care to the population served by this service. It can thus be seen that leadership in APH promotes optimization in the environment and organization of the nursing work process, ensuring that nurses have the power to develop their practices in a more comprehensive scope and thus offer high-quality care.

Therefore, according to this type of leadership, a good leader has the ability to: build trusting relationships and more productive work environments; encourage the autonomy of their professionals; share power; and, consequently, achieve greater excellence in patient care and safety. Leadership competence in nursing has a major influence on professional practice, especially when structural empowerment is analyzed. Less structurally empowered nurse leaders are more likely to become frustrated when trying to improve their professional practice and innovate in patient care^(10,11)^.

An integrative review^(12)^, published in a prominent international journal, shows that Coachin*g* Leadership is still an emerging area of nursing research. Furthermore, the authors of the study point out that the development of research based on the Coaching Leadership framework can result in subsidies for health services to invest in the dimensions of the coaching process, in order to build the empowerment of nurses and, consequently, provide the necessary skills for these professionals to perform their work at the highest levels.

The findings of new studies on the subject provide an opportunity to further explore the benefits, challenges, and ways in which Coaching Leadership can be implemented to support nurses in their development and practice. Thus, when analyzing the context of fixed and mobile APH, research exploring the interfaces of this leadership model established by nurses with other variables^(4,9)^, such as empowerment^(13)^, is still in its infancy. For the present study, Kanter’s structural empowerment was adopted, which understands power as the ability to take action and mobilize and employ the resources necessary to achieve organizational goals^(10)^.

As a hypothesis, this article sought to test whether nurses’ self-perception of Coaching Leadership correlates with structural empowerment, considering the context of mobile and fixed APH. Given the above, this article sought to analyze the correlation between Coaching Leadership exercised by mobile and fixed APH nurses and the structural empowerment of these professionals.

## METHOD

### Type of Study

This is a descriptive, analytical study that aims to examine the relationship between the variables of Coaching Leadership and the structural empowerment of nurses working in mobile and fixed APH. Furthermore, this research followed the recommendations of Strengthening the Reporting of Observational Studies in Epidemiology (STROBE)^(14)^ to support the communication of findings and steps taken.

### Study Location

The study setting corresponded to the RUE present in a metropolitan capital in the southeastern region of the country. It should also be noted that the organization of APH services (mobile and fixed) in this municipality consists of the SAMU center, six decentralized bases, and nine UPAs. Considering the latter units, emails were sent to the general and nursing coordinators, and three of the nine UPAs did not respond to the emails for data collection.

### Research Subjects

At the time of the research, the total number of nurses in the units (SAMU and UPA) was 258 professionals, both in the care and management areas. The sample was established for convenience, that is, non-probabilistic, with nurses who performed care or management activities in the analyzed units, within the established inclusion criteria.

Nurses who had been in the position for at least six months were included in the research, in order to ensure that the worker already felt comfortable and capable of evaluating the practice of Coaching Leadership and structural empowerment (39 professionals did not meet this criterion). Regarding the exclusion criteria, professionals in the above-mentioned category who were on vacation or bonus leave (n = 34), or on leave for health reasons (n = 10), did not participate in the study. It should be noted that 21 professionals refused to participate in the study, resulting in a total of 154 nurses eligible to participate in the study.

Based on the 154 professionals, the sample calculation was performed, adopting as parameters a sampling error of 8%, a confidence level of 95%, and a more homogeneous population distribution, totaling a minimum number of 61 nurses as representation of the population. The number of respondents (79 nurses) was above that established by the sample calculation. The calculation was not stratified for the units (mobile and fixed APH), as the aim was to analyze the overall context of the APH. It should be noted that the following were considered as sample loss: professionals who, after two attempts, were not located on their respective shifts and those who did not return the completed questionnaires or returned them blank.

### Instruments Used in Data Collection

Three questionnaires were applied, namely: the characterization of the subjects, containing sociodemographic and profession-related data; the Nurse Self-Perception Questionnaire in the Exercise of Leadership (QUAPEEL in the Portuguese acronym)^(8)^; and the cross-culturally validated questionnaire for Brazil, Conditions of Effectiveness at Work II (CET-II in the Portuguese acronym), which analyzes structural empowerment^(15)^.

The sociodemographic and profession-related data were: gender (male and female); education (Specialization, Master in Business Administration (MBA), Master’s Degree, Doctorate; Complementary Course – Basic Life Support, etc.); type of employment relationship (statutory public service, private law contract, fixed-term contract, and others); having another employment relationship; having already held a managerial position; age (calculation of the difference between the dates of data collection and birth); length of education (difference between the dates of data collection and graduation); and length of service at the unit (time elapsed between the dates of data collection and admission to the unit).

QUAPEEL, which was developed and validated in Brazil, has structured questions composed of three parts: the first corresponds to the sociodemographic data of the subjects; the second consists of questions related to the subjects’ knowledge of leadership; and the third presents questions related to the skills and attitudes exercised by leaders in the practice of Coaching Leadership through the self-perception of nurses^(8)^. It should be emphasized that only the third part of the questionnaire was used for this research.

This questionnaire consists of 20 items, equally subdivided into four domains: Communication; Giving and receiving feedback; Giving and exercising influence; and Supporting the team to achieve results. The questionnaire uses a Likert scale, with the following configuration for each item: Never (1); Rarely (2); Not always (3); Almost always (4); and Always (5). The item “Not Applicable” (NA), provided in the Likert scale, corresponds to the scenario in which the professional believes they do not perform such an action; for this, the value zero (0) was assigned. Therefore, the overall score of the instrument varies between 0 and 100, i.e., values close to 0 correspond to the lowest perception of Coaching Leadership practice, while scores close to 100 correspond to the highest perception of this type of leadership^(8)^.

The Conditions of Effectiveness at Work II (CET-II), a cross-culturally adapted version, was used to analyze structural empowerment in the work environment of APH nurses^(15)^. The original instrument was designed and developed by Canadian researchers, with Kanter’s empowerment as its theoretical reference^(16)^.

In Brazil, the adaptation and cross-cultural validation of this instrument was carried out by a group of researchers from Paraná, with the aim of evaluating the work of nurses in different settings in relation to structural empowerment conditions. It should be reiterated that this questionnaire is widely used by researchers in the field and, in the cross-cultural validation process, obtained a Cronbach’s alpha of 0.88, representing high internal consistency among the internal responses^(15)^.

The CET-II is structured in 20 items, divided into six dimensions: Timeliness; Information; Support; Resources; Formal Power; and Informal Power. There is also a construct that encompasses a broader perspective of empowerment, containing two items to analyze global empowerment, but these were not applied in the present study, as it only analyzed the six dimensions mentioned above^(16)^.

The CET-II questionnaire is measured using a Likert scale, with scores ranging from 1 (none) to 5 (very), while values between 2 and 4 represent intermediate values. The items in each component are added together and then averaged to provide a score ranging from 1 to 5 for each dimension, as mentioned above. The sum of the dimensions can vary from 6 to 30, with values between 6–13 meaning low levels of empowerment; 14–22 meaning moderate levels of empowerment; and 23–30 meaning high levels of empowerment^(15,16)^.

### Data Collection and Analysis

A group consisting of a research professor and four students was responsible for delivering the questionnaires to the subjects who agreed to participate in the study. To this end, the administrative and nursing coordinators of the units in question were contacted in advance so that the questionnaires could be delivered to the nurses in person, and the collection of the instruments was scheduled.

The data collected through the questionnaires were double- entered into spreadsheets and compared, thus ensuring that there were no typing errors. Descriptive statistical analysis and correlation between variables were then performed. For this purpose, the statistical program R version 4.3.3 was used for data processing. Qualitative variables were summarized in simple and relative frequencies using percentages. Quantitative variables were organized by means, medians, standard deviation (SD), first and third *quartiles*, and minimum and maximum values.

Initially, the Shapiro-Wilk normality test was used to evaluate the distribution of the variables. Pearson’s correlation, which can be positive or negative, was applied to analyze the correlation between self-perception of Coaching Leadership and structural empowerment. A significance level of 5% was used for all statistical tests (α = 0.05). In addition, the correlations were interpreted/evaluated according to the size of the correlation, positive or negative, and could thus present: very high correlation (values greater than 0.9 to 1.0; or greater than –0.9 to –1.0); high correlation (values greater than –0.7 to –0.9; or greater than 0.7 to 0.9); moderate correlation (values greater than 0.5 to 0.7 or greater than –0.5 to –0.7); low correlation (values greater than 0.3 to 0.5 or values greater than –0.3 to –0.5) and; insignificant correlation (values above 0.0 to 0.3 or greater than 0.0 to –0.3)^(17)^.

### Ethical Aspects of the Research

The research was reviewed and cleared by the Research Ethics Committees of the Federal University of Minas Gerais, under number 5.904. 124 (on February 20, 2023), and by the Municipal Health Secretariat of Belo Horizonte, under number 6.063.014 (on May 16, 2023), in order to comply with the provisions of Resolution No. 466/2012 of the National Health Council. In addition, all participants were briefed on the research and signed the Free and Informed Consent Form before completing the questionnaires.

## RESULTS

The number of nurses participating in the research during the data collection period was 79 professionals, which corresponded to 48.17% of eligible participants. Regarding sample losses: 22 nurses were not located on their respective shifts after two unsuccessful attempts; and 53 professionals did not complete or return the questionnaires. The data corresponding to the characterization of nurses in terms of sociodemographic or profession-related variables are shown in Tables 1 and 2.

**Table 1:** Characterization of nurses from APH units (mobile and fixed) according to the variables: gender, education (specialization, MBA, master’s degree, doctorate, complementary course), type of employment contract, having another employment contract, and experience in a managerial position – Belo Horizonte, MG, Brazil, 2023 (N = 79).

Variables	Description	N	Percentage (%)
Gender	Feminine	61	77.21
	Masculine	18	22.79
Specialization	No	9	11.39
	Yes	70	88.61
MBA	No	74	93.67
	Yes	5	6.33
Masters’ degree	No	69	87.34
	Yes	10	12.66
Doctorate	No	78	98.73
	Yes	1	1.27
Complementary course	No	42	53.16
	Yes	37	46.84
Working status	Private contract	18	22.79
	Public contract	35	44.30
	Fixed-time contract	26	32.91
Other jobs	No	39	49.37
	Yes	40	50.63
Managerial experience	No	49	62.02
	Yes	30	37.98

Source: Authors.

**Table 2:** Characterization of nurses in APH units (mobile and fixed) according to the variables: age, length of training, and length of service at the institution – Belo Horizonte, MG, Brazil, 2023 (N = 79).

Variables	Average	Standard deviation	Minimum (years)	Maximum (years)
Age	41.10	8.12	24	68
Length since graduated	13.66	7.32	1	34
Length of service	5.95	1.32	6 months	26

Source: Authors.

Table 1 shows that there is a significant number of female nurses (n = 61; 77.22%). Most of the professionals who participated in the survey reported having specialization (n = 70; 88.61%), having statutory employment (n = 35; 44.30%), having other employment (n = 40; 50.63%), and no experience in a managerial position (n = 49; 62.02%). Table 2 shows that, on average, the professionals were 41.1 years old (SD = 8.12), had been trained for 13.66 years (SD = 7.32), and had been working at the institution for 5.95 years (SD = 1.32).

Table 3 shows the correlation analyses between the domains of the instruments used in this study. Although there was a low correlation between the total QUAPEEL and CET-II scores and between the domains of the instrument, it is noted that Coaching Leadership influences the empowerment of nurses working in the APH context, a fact confirmed by a significant number of correlations between the total QUAPEEL score and the domains of the instrument that measures structural empowerment (information, support, resources, and formal power).

**Table 3:** Correlation between the practice of Coaching Leadership and the structural empowerment of nurses in APH units – Belo Horizonte, MG, Brazil, 2023 (N = 79).

Dominions	Timeliness	Information	Support	Resources	Formal power	Informal power	CET-II total score
Communication	0.317*	**0.258**	**0.326**	**0.477**	**0.353**	**0.262**	**0.467**
	0.004**	**0.022**	**0.003**	**<0.001**	**0.001**	**0.020**	**<0.001**
Give/receive feedback	**0.227**	**0.221**	**0.278**	**0.366**	**0.360**	0.062	**0.366**
	**0.045**	**0.050**	**0.013**	**<0.001**	**0.001**	0.584	**<0.001**
Give/exert influence	0.113	0.201	0.147	0.165	**0.246**	–0.129	0.189
	0.321	0.076	0.196	0.145	**0.029**	0.258	0.096
Support the team in achieving results	0.122	0.202	**0.246**	0.205	**0.314**	0.010	**0.272**
	0.286	0.074	**0.029**	0.069	**0.005**	0.928	**0.015**
**QUAPEEL total score**	0.216	**0.266**	**0.298**	**0.340**	**0.386**	0.037	**0.376**
	0.056	**0.018**	**0.008**	**0.002**	**<0.001**	0.747	**<0.001**

Source: Authors;

*Pearson correlation;

**p-value.

In a more detailed analysis of the QUAPEEL domains, “communication” was the one that correlated with all CET-II domains, including the total score of the instrument, which demonstrates the relevance of this competence for nurse leadership in this scenario. Next, “giving and receiving feedback” was the second construct to influence structural empowerment, showing no correlation between this domain of QUAPEEL and informal power present in CET-II alone. “Giving and exercising influence” was the domain with the lowest correlation with the constructs of the structural empowerment instrument, as it showed a correlation only with formal power, thus denoting a competency to be improved by professionals.

## DISCUSSION

Regarding the characteristics of the participants, most of the professionals were female nurses with specialization, with an average age of 41.1 years, 13.66 years of training, and 5.95 years of experience at the institution. These findings are similar to those of three national studies that applied QUAPEEL in the context of APH^(4)^ and CET-II in the context of hospital urgency/emergency^(18,19)^.

However, the result related to having another job was different from that found in a study conducted at a SAMU in the Federal District, where the percentage was higher for people without a second job^(20)^. Not having experience in a managerial position and having a statutory employment relationship resulted in a percentage close to that of two studies in the context of national urgent and emergency care, one in the state of Goiás^(9)^ and the other in the metropolitan region of Belo Horizonte^(18)^.

The present study found that the majority of nurses had no experience in management positions. In view of this, it should be noted that emergency nurses, especially those who already have management experience, are able to respond and adapt effectively to the current and future challenges of care management practices^(6)^.

In this study, it was evident that the total QUAPEEL score had a weak positive correlation with virtually all constructs and with the total CET-II score (R = 0.376; p-value = <0.001). It should be noted that, in this way, the role of nursing leadership styles and models favors the empowerment of these professionals.

Regarding this relationship, two recent articles^(20,21)^, in the context of urgent and emergency care, express the potential of this competence, in different models and styles, on the elements of structural empowerment. The study conducted in Jordan observed that clinical leadership practices showed a significant positive relationship with structural empowerment (r = 0.65; P < 0.01)^(21)^; in turn, nurses in the emergency department in Nepal, who exercised collaborative leadership to empower their teams, achieved greater adherence and better responses to training on initial trauma care^(22)^.

The experiences of nursing staff can be directly affected by the way nurses exercise their leadership and establish favorable working conditions for the development of their team. From this perspective, a Brazilian systematic review^(23)^ presents evidence of how leadership impacts structural empowerment, consequently influencing job satisfaction, engagement, and increased quality of care provided^(23)^.

Although the total QUAPEEL score did not correlate with the “Timeliness” domain of CET-II, an integrative review on empowerment in the context of urgent and emergency care indicates that in order to achieve autonomy at work and better levels of self-efficacy through opportunities, it is essential that nurses are grounded in positive leadership models such as resonant and clinical leadership^(13)^. Furthermore, the increase in opportunities for these professionals in the RUE can be seen in Advanced Practice Nursing, which represents the growth and expanded role of nurses in Brazilian APH^(24)^.

Table 3 shows how “communication” in nurses’ self-perception is a significant component in the Coaching Leadership model, as it significantly influences their empowerment. Communication by nurse leaders is crucial for building good relationships and empowering team members. To this end, it must be based on the pillars of communication: a supportive rather than imposing dialogue; sharing the leader’s visions and values; and communication and support for evidence- based practices^(25)^.

From the perspective of those that are led, two studies^(4,9)^ that analyzed the perception of nursing technicians on the exercise of Coaching Leadership by their coordinators in the mobile APH context, “communication” was the second domain with the highest average, correlating with the shortest training time^(4)^ and job satisfaction^(9)^. Communication in Coaching Leadership, according to a recent integrative review^(12)^, has a significant impact on the individual support of led nursing professionals, which is reflected in the development of new skills in these professionals that enable them to see new paths for teamwork^(12)^.

“Giving and receiving feedback” from the QUAPEEL instrument was not correlated with informal power, a domain of CET, which demonstrates the relevance of this instrument in the empowerment process. In this sense, two studies converge on this result^(12,26)^. The first analyzes the leadership of advanced practice nurses and revealed that this competency, when based on the coaching process, promotes empowerment and improves group performance, which is why it is evident that nurses need to value feedback mechanisms^(26)^.

The second study, an integrative review^(12)^, emphasized feedback in coaching leadership as a facilitator for the empowerment of nurses. With this, health organizations and their professionals can perform at a high level and meet institutional standards and protocols^(12)^. Nurses need to be trained and spend adequate/appropriate time on feedback, especially when addressing issues such as errors. Thus, leaders must be able to offer the necessary support and promote a culture of non-punishment, in addition to systematically analyzing failures^(25)^.

Formal power, according to the structural empowerment framework, comprises aspects related to the hierarchical position that an individual has within an organization^(10)^. This construct of the CET-II instrument was the only one that correlated with the dimension of Coaching Leadership “giving and exercising influence,” as shown in Table 3.

The influence of nurses is a skill to be encouraged in professionals at the APH units studied, given the results of the correlations. This finding shows that the ability to inspire and motivate teams, combined with effective communication skills and quick decision-making, are fundamental to ensuring the quality of care and patient safety in this dynamic and challenging context that comprises urgent and emergency care^(6,24)^.

Although the dimension “Supporting the team to achieve results” showed a lower correlation with the CET-II domains, a recent study^(5)^ of the urgent and emergency care network of a municipality in São Paulo pointed to the need for the nurse leader to build credibility in order to obtain support from all levels of practice, as well as in all scenarios. Considering that SAMU and UPAs are part of the REU context, in order to empower themselves, nurses need to understand internal and external policies, critically analyze emerging demands, and recognize the space and opportunities for leadership for others^(5)^.

The limitation of the research was mainly related to the number of professionals who were not found on duty to be invited to participate in the research. Even so, this limitation did not strongly impact the statistical analyses, nor did it minimize the relevance and innovative nature of the research by presenting an investigation into the leadership of nurses not only in the context of mobile APH, but also in Fixed Emergency Care Units, in the quality of UPAs. In addition, the study also made it possible to analyze the correlation between two variables that are relevant to the professional practice of emergency and urgent care nurses.

The contributions of this research demonstrate the importance of Coaching Leadership in empowering nurses working in the context of mobile and fixed APH, with emphasis on the positive correlation between “communication” and all domains of structural empowerment. This finding has direct implications for professional practice, as it suggests that the development of communication skills by nurses can strengthen their ability to influence and lead the team more effectively, improving the organization and outcomes of care. In addition, the relationship obtained between “giving and receiving feedback” and structural empowerment reinforces the need for evaluation practices and continuous support among professionals.

## CONCLUSION

The findings of this article demonstrate that the Coaching Leadership exercised by mobile and fixed APH nurses has a positive correlation with structural empowerment. This aspect was observed by the correlation between the total scores of the instruments used in the research, as well as by the numerous correlations between the domains of QUAPEEL and CET-II. Thus, these findings guide training strategies focused on elements and competencies such as communication and feedback, which have a positive impact on the quality of care, patient satisfaction, and the continuous professional development of nurses in the APH.

## Data Availability

The entire dataset supporting the results of this study was published in the article itself.
