# Supplementary material for: Coaching Leadership and structural empowerment of nurses in mobile and fixed Pre-Hospital Care
Source: Rev Esc Enferm USP. 2026 Jan 9;59:e20240450. doi: 10.1590/1980-220X-REEUSP-2024-0450en (PMC12794808; doi:10.1590/1980-220X-REEUSP-2024-0450en)
Supplement: Translation

# Translation: Liderança *Coaching* e empoderamento estrutural dos enfermeiros do Atendimento Pré-Hospitalar móvel e fixo

**DOI:** 10.1590/1980-220X-REEUSP-2024-0450pt

**Authors:** Letícia Santiago Utsch, Fernanda Oliveira Santos, Katharina Júlia Lacerda Oliveira, Hercules de Oliveira Carmo, André Almeida de Moura

**Affiliations:** 1 Universidade Federal de Minas Gerais, Escola Enfermagem, Belo Horizonte, MG, Brasil.; 2 Universidade de São Paulo, Escola de Enfermagem, Departamento de Orientação Profissional, São Paulo, SP, Brasil.

**Keywords:** Liderança, Empoderamento, Enfermagem em Emergência, Enfermeiras e Enfermeiros, Serviços Médicos de Emergência

## Abstract

**Objetivo:**

Analisar a correlação entre a Liderança *Coaching* exercida pelos enfermeiros do Atendimento Pré-Hospitalar (APH) móvel e fixo com o empoderamento estrutural desses profissionais.

**Método:**

Estudo analítico e descritivo com 79 enfermeiros do pré-hospitalar móvel e fixo. Utilizaram-se os questionários: Caracterização dos sujeitos; Questionário de Autopercepção do Enfermeiro no Exercício da Liderança; e Questionário de Condições de Eficácia no Trabalho II (CET-II). Empregaram-se a análise descritiva e a análise inferencial com o uso do teste de Spearman para determinar as correlações entre as dimensões do instrumento, adotando-se nível de significância de 5%.

**Resultados:**

A Liderança *Coaching* influencia positivamente o empoderamento dos enfermeiros atuantes no contexto do APH evidenciado pela correlação fraca entre escores totais dos instrumentos aplicados na pesquisa (R = 0,376; *p*-valor = <0,001). “Comunicação” correlacionou-se com todos os domínios do CET-II, a incluir o “Escore total” do instrumento.

**Conclusão:**

É importante que as organizações de APH móvel e fixo invistam na liderança dos seus enfermeiros, a partir da utilização do modelo de Liderança *Coaching*, tendo em vista especialmente que ela tem uma influência positiva sobre o empoderamento do enfermeiro.

## INTRODUÇÃO

A atuação do enfermeiro no serviço de urgência e emergência é de grande relevância, haja vista que esse profissional está diretamente em contato com o paciente, desde o primeiro momento (no local da cena) por via do Atendimento Pré-Hospitalar (APH) móvel, na qualidade do Serviço de Atendimento Móvel de Urgência (SAMU), perpassando pelo pré-hospitalar fixo que compreendem as Unidades de Pronto Atendimento (UPA), até o hospital que acolhe e presta cuidados intra-hospitalares às necessidades agudizadas de saúde. Todos esses cenários de cuidado demandam do enfermeiro atitudes, habilidades, conhecimentos técnico-científico, além da observância de aspectos éticos relacionados à urgência e emergência^(1)^.

No Brasil, a Rede de Atenção às Urgências e Emergências (RUE) tem como finalidade desenvolver ações de promoção, prevenção e vigilância à saúde, bem como atendimentos e cuidados aos usuários em condições agudas. Diante desse cenário, o processo de trabalho na urgência demanda um dinamismo significativo por parte dos profissionais em resposta às altas demandas, aos eventos inesperados, à capacitação profissional constante, à sinergia e à intercomunicação na equipe, tanto no pré, como no intra-hospitalar, para a promoção de um atendimento seguro e com qualidade^(2)^.

Assim, considerando-se essa dinamicidade, os enfermeiros desempenham papéis imprescindíveis nesse atendimento, principalmente no emprego da liderança na sua prática profissional. É por intermédio dessa competência que tal profissional alcança melhores resultados para pacientes e equipes e promove a interlocução com os demais serviços e setores de saúde^(3,4)^. Logo, percebe-se que a liderança do enfermeiro nesse contexto busca favorecer as dimensões do seu processo de trabalho^(5)^.

Editorial recente^(6)^, de um periódico internacional de enfermagem em emergências, apresenta reflexão sobre a liderança enquanto competência fundamental para a prática do enfermeiro no setor de emergência e sublinha que, para sua efetiva mobilização, esse profissional deve atualizar-se constantemente e associar à liderança o uso de outras competências e de ferramentas gerenciais. Sendo assim, para que o enfermeiro possa exercer essa competência, o referido profissional deverá transpor alguns desafios como a sobrecarga de trabalho e de demanda, valendo-se assim de estratégias para superação dos mesmos. Para tanto, o texto aponta que além da liderança, o enfermeiro requer domínio de uma ampla gama de ferramentas como: pensamento estratégico, resolução de problemas, inteligência emocional, colaboração, formação de equipe e tomada de decisão.

Corroborando esse editorial, estudo etnográfico com enfermeiros do serviço pré-hospitalar sueco afirma que, a partir da sua responsabilidade e da sua liderança, o enfermeiro tem um papel primordial de elucidar os valores dos liderados, incentivando-os a desenvolverem suas próprias qualidades, promovendo, consequentemente, ambiente de prática favorável na equipe de enfermagem^(7)^.

Diante da magnitude da liderança para atuação do enfermeiro na urgência, aponta-se o modelo da Liderança *Coaching* como uma referência para o exercício dessa competência pelos enfermeiros. Conceitualmente, a Liderança *Coaching* baseia- se no processo *coaching* que consiste em uma competência a partir da qual o líder busca influenciar a equipe para alcance dos objetivos, na mesma medida que possibilita o desenvolvimento dos seus liderados. Para tanto, esse modelo fundamenta-se em quatro dimensões do processo *coaching*, a citar: “Comunicação”, “Dar e receber feedback”, “Dar poder e exercer influência” e “Apoio à equipe”^(4,8)^.

Baseado nesse modelo de liderança no APH, dois artigos brasileiros^(4,9)^, desenvolvidos no Estado de Goiás, revelaram que os enfermeiros ao desempenharem a Liderança *Coaching* promovem transformações positivas no seu espaço de trabalho como a satisfação dos seus liderados e, da mesma forma, possibilitam a prestação de cuidado com qualidade e segurança para a população atendida por esse serviço. Nota-se, dessa maneira, que a liderança no APH promove uma otimização no ambiente e na organização do processo de trabalho da enfermagem, de maneira a garantir que os enfermeiros tenham poderes para desenvolverem suas práticas em um escopo mais completo e, assim, oferecerem atendimento de alta qualidade.

Portanto, de acordo com esse tipo de liderança, um bom líder tem a capacidade de: construir relações de confiança e ambientes de trabalho mais profícuos; estimular a autonomia dos seus profissionais; compartilhar o poder e obter, por conseguinte, maior excelência no atendimento e segurança do paciente. A competência da liderança na enfermagem tem grande influência sobre a prática profissional, especialmente quando se analisa o empoderamento estrutural. Enfermeiros líderes menos empoderados estruturalmente apresentam maiores chances de tornarem-se mais frustrados ao tentarem melhorar a sua prática profissional e inovar no atendimento ao paciente^(10,11)^.

Revisão integrativa^(12)^, publicada em expressivo periódico internacional, evidencia que a Liderança *Coaching* ainda é uma área emergente da pesquisa em enfermagem. Ademais, os autores do trabalho sinalizam que o desenvolvimento de pesquisas baseada no referencial da Liderança *Coaching* pode resultar em subsídios para que os serviços de saúde invistam nas dimensões do processo *coaching*, de maneira a construir o empoderamento dos enfermeiros e, por conseguinte, oportunizar as competências necessárias para que esse profissional desenvolva seu trabalho nos mais altos níveis.

Os achados de novos trabalhos na temática estabelecem-se como uma oportunidade de explorar mais os benefícios, os desafios e as maneiras pelas quais a Liderança *Coaching* pode ser implementada para apoiar enfermeiros em seu desenvolvimento e sua prática. Sendo assim, ao analisar o contexto do APH fixo e móvel, ainda são incipientes as pesquisas que explorem as interfaces desse modelo de liderança estabelecidas pelos enfermeiros com outras variáveis^(4,9)^, a exemplo do empoderamento^(13)^. Para o presente, adotou-se o empoderamento estrutural de Kanter, que compreende o poder como capacidade de fazer as ações e mobilizar e empregar recursos necessários para atingir as metas organizacionais^(10)^.

Como hipótese, o presente artigo buscou testar se a autopercepção dos enfermeiros acerca da Liderança *Coaching* correlaciona-se com empoderamento estrutural, tendo em vista o contexto do APH móvel e fixo. Diante do exposto, o presente artigo buscou analisar a correlação entre a Liderança *Coaching* exercida pelos enfermeiros do APH móvel e fixo com o empoderamento estrutural desses profissionais.

## MÉTODO

### Tipo de Estudo

Trata-se de um estudo descritivo, analítico, com vistas a examinar a relação entre as variáveis da Liderança *Coaching* e o empoderamento estrutural dos enfermeiros atuantes no APH móvel e fixo. Ainda, a presente pesquisa seguiu as recomendações do *Strengthening the Reporting of Observational Studies in Epidemiology* (STROBE)^(14)^ para subsidiar a comunicação dos achados e das etapas percorridas.

### Local de Estudo

O cenário do estudo correspondeu à RUE presente em uma capital metropolitana da região sudeste do país. Cabe destacar, outrossim, que a organização dos serviços de APH (móvel e fixo) nesse município está constituída pela central do SAMU, seis bases descentralizadas, além das nove UPA. Considerando-se essas últimas unidades, foram enviados e-mails para as coordenações gerais e de enfermagem e três das nove UPA não responderam aos e-mails para realização da coleta de dados.

### Sujeitos da Pesquisa

No momento da realização da pesquisa, o total de enfermeiros das unidades (SAMU e UPA) era de 258 profissionais, tanto no âmbito assistencial quanto do gerenciamento. Estabeleceu-se a amostra por conveniência, ou seja, não probabilística com os enfermeiros que realizavam ações assistenciais ou atividades gerenciais nas unidades analisadas, dentro dos critérios de inclusão estabelecidos.

Foram inclusos na pesquisa os enfermeiros no exercício do cargo há pelo menos seis meses, de modo a garantir que o trabalhador já se sentia ambientado e capaz de avaliar a prática da Liderança *Coaching* e o empoderamento estrutural (39 profissionais não atendiam a esse critério). No que tange aos critérios de exclusão, não participaram da pesquisa os profissionais da categoria supracitada que se encontravam em uso do direito de férias ou de licença-prêmio (n = 34), ou então afastados por motivos de saúde (n = 10). Cabe apontar que 21 profissionais se recusaram participar da pesquisa, o que totalizou 154 enfermeiros elegíveis para participarem da pesquisa.

Com base nos 154 profissionais, realizou-se o cálculo amostral, adotando-se como parâmetros um erro amostral de 8%, nível de confiança de 95% e uma distribuição da população mais homogênea, totalizando-se o número mínimo de 61 enfermeiros como representação da população. O número de respondentes (79 enfermeiros) foi acima do estabelecido pelo cálculo amostral, O cálculo não foi estratificado para as unidades (APH móvel e fixo), pois buscava-se a análise global do contexto do APH. Ressalta-se, apenas, que foram considerados como perda da amostra: os profissionais que, após duas tentativas, não foram localizados nos seus respectivos plantões e os aqueles que não devolveram os questionários preenchidos ou os tenham devolvido em branco.

### Instrumentos Utilizados na Coleta de Dados

Foram aplicados três questionários, sendo eles: a caracterização dos sujeitos que contém os dados sociodemográficos e os relacionados à profissão; o Questionário de Autopercepção do Enfermeiro no Exercício da Liderança (QUAPEEL)^(8)^ e; o questionário, validado transculturamente para o Brasil, de Condições de Eficácia no Trabalho II (CET-II) que analisa o empoderamento estrutural^(15)^.

Os dados sociodemográficos e relacionados à profissão foram: sexo (masculino e feminino); formação (Especialização, *Master in Business Administration* MBA, Mestrado, Doutorado; Curso complementar – Suporte Básico de Vida etc.); tipo de vínculo empregatício (estatutário, celetista, contrato por tempo determinado e outros); possuir outro vínculo empregatício; já ter assumido cargo gerencial; idade (cálculo da diferença entre as datas de coleta e de nascimento); tempo de formação (diferença entre as datas de coleta e de conclusão da graduação) e; tempo de atuação na unidade (tempo decorrido entre as datas de coleta e de admissão na unidade).

O QUAPEEL, que foi construído e validado no Brasil, dispõe de questões estruturadas e compostas por três partes: a primeira corresponde aos dados sociodemográficos dos sujeitos; a segunda consiste em perguntas relativas aos conhecimentos dos sujeitos sobre a temática da liderança, enquanto a terceira apresenta questões relacionadas às habilidades e às atitudes exercidas pelos líderes na prática da Liderança *Coaching* por meio da autopercepção do enfermeiro^(8)^. Enfatiza-se que para a presente pesquisa foi utilizada apenas a terceira parte do questionário.

Esse questionário é composto por 20 itens, igualmente subdivididos em quatro domínios: Comunicação; Dar e receber feedback; Dar e exercer influência; Apoiar a equipe para alcance de resultados. O questionário utiliza-se de uma escala Likert, com a seguinte configuração para cada item: Nunca (1); Raramente (2); Nem sempre (3); Quase sempre (4) e; Sempre (5). O item “Não se Aplica” (NA), disposto na escala de Likert, corresponde ao cenário em que o profissional acredita não desenvolver tal ação; para isso, foi atribuído o valor de zero (0). Portanto, a variação do escore do geral do instrumento está entre os valores de 0 a 100, ou seja, os valores que se aproximam de 0 correspondem à menor percepção da prática de Liderança *Coaching*, ao passo que, nas pontuações perto de 100, maior é a percepção da prática desse tipo de liderança^(8)^.

O Questionário de Condições de Eficácia no Trabalho II (CET-II), versão adaptada transculturamente, foi empregado com o objetivo de analisar o empoderamento estrutural no ambiente de trabalho dos enfermeiros do APH^(15)^. O instrumento original teve sua concepção e desenvolvimento por pesquisadores canadenses, tendo como referencial teórico o empoderamento de Kanter^(16)^.

No Brasil, a adaptação e a validação transcultural desse instrumento foi realizada pelo grupo de pesquisadores do Paraná, com a finalidade de avaliar o trabalho do enfermeiro em diferentes cenários, com relação às condições de empoderamento estruturais. Reitera-se que o presente questionário é amplamente utilizado pelos pesquisadores da área e, no processo de validação transcultural, obteve alfa de Cronbach de 0,88, representando elevada consistência interna entre as respostas^(15)^.

O CET-II está estruturado em 20 itens, divididos em seis dimensões: Oportunidade; Informação; Suporte; Recursos; Poder formal e Poder informal. Há ainda um constructo que engloba uma perspectiva mais ampla do empoderamento, contendo dois itens para analisar o empoderamento global, mas que não foram aplicados na presente pesquisa, pelo fato de analisar apenas as seis dimensões referidas anteriormente^(16)^.

A mensuração do questionário CET-II é feita por uma escala do tipo Likert, cuja pontuação varia de 1 (nenhum/nenhuma) a 5 (muito/muita), enquanto valores entre 2 e 4 representam valores intermediários. Os itens de cada um dos componentes são somados e, na sequência, calculada uma média, com vistas a fornecer uma pontuação que varia entre 1–5 para cada dimensão, como mencionado anteriormente. A soma das dimensões poderá variar de 6 a 30, sendo que valores entre: 6-13 significam baixos níveis de empoderamento; 14–22 significam níveis moderados de empoderamento e; 23-30 altos níveis de empoderamento^(15,16)^.

### Coleta e Análise Dos Dados

Um grupo constituído por um docente pesquisador e quatro alunas foi responsável por entregar os questionários para os sujeitos que consentissem participar da pesquisa. Para tanto, previamente foi realizado o contato com os coordenadores administrativos e de enfermagem das unidades em questão para que fossem entregues os questionários aos enfermeiros pessoalmente, bem como o agendamento do recolhimento dos instrumentos.

Os dados coletados por intermédio dos questionários foram duplamente digitados em planilhas e confrontados, assegurando-se, dessa forma, não haver erros de digitação, sendo posteriormente realizadas as análises estatísticas descritiva e de correlação entre as variáveis. Para isso, empregou-se o programa estatístico R versão 4.3.3 para o tratamento dos dados. As variáveis qualitativas foram resumidas em frequências simples e relativas utilizando-se para tanto as porcentagens. Já as variáveis quantitativas foram organizadas por médias, medianas, desvio-padrão (DP), primeiro e terceiro *quartis* e valores mínimos e máximos.

Inicialmente, foi utilizado o teste de normalidade Shapiro-Wilk, de maneira a possibilitar a avaliação da distribuição das variáveis. Para a análise de correlação entre a autopercepção da Liderança *Coaching* e o empoderamento estrutural foi aplicada a correlação de Pearson, que pode apresentar-se positiva ou negativamente. Empregou-se o nível de significância de 5% para todos os testes estatísticos (α = 0,05). Complementarmente, as correlações foram interpretadas/avaliadas conforme o tamanho da correlação, positiva ou negativamente, podendo assim apresentar: correlação muito alta (valores maiores que 0,9 até 1,0; ou maiores que –0,9 até –1,0); correlação alta (valores maiores que 0,7 até 0,9; ou maiores que –0,7 até –0,9); correlação moderada (valores maiores que 0,5 até 0,7 ou maiores que –0,5 até –0,7); baixa correlação (valores maiores que 0,3 até 0,5 ou valores maiores que –0,3 até –0,5) e; correlação insignificante (valores acima de 0,0 até 0,3 ou maiores que 0,0 até 0,3)^(17)^.

### Aspectos Éticos da Pesquisa

A pesquisa foi apreciada e aprovada pelos Comitês de Ética em Pesquisa da Universidade Federal de Minas Gerais, sob o número 5.904.124 (em 20 de fevereiro de 2023), e da Secretaria Municipal de Saúde de Belo Horizonte, sob número 6.063.014 (em 16 de maio de 2023), de maneira a atender às determinações da Resolução nº 466/2012 do Conselho Nacional de Saúde. Além disso, todos os participantes foram orientados sobre a pesquisa e assinaram o Termo de Consentimento Livre e Esclarecido antes de aplicarem os questionários.

## RESULTADOS

O número de enfermeiros participantes da pesquisa no período da coleta dos dados foi de 79 profissionais, o que correspondeu ao percentual de 48,17% dos participantes elegíveis. No que tange às perdas da amostra: 22 enfermeiros não foram localizados nos seus respectivos plantões, após duas tentativas sem sucesso e; 53 profissionais não preencheram ou devolveram os questionários em branco. Os dados correspondentes à caracterização dos enfermeiros quanto às variáveis sociodemográficas ou relacionadas à profissão estão dispostos nas Tabelas 1 e 2.

**Tabela 1:** Caracterização dos enfermeiros das unidades de APH (móvel e fixo) quanto às variáveis: sexo, formação (especialização, MBA, mestrado, doutorado, curso complementar), tipo de vínculo, possuir outro vínculo empregatício e experiência em cargo gerencial – Belo Horizonte, MG, Brasil, 2023 (N = 79).

Variáveis	Descrição	N	Porcentagem (%)
Sexo	Feminino	61	77,21
	Masculino	18	22,79
Especialização	Não	9	11,39
	Sim	70	88,61
MBA	Não	74	93,67
	Sim	5	6,33
Mestrado	Não	69	87,34
	Sim	10	12,66
Doutorado	Não	78	98,73
	Sim	1	1,27
Curso complementar	Não	42	53,16
	Sim	37	46,84
Tipo de vínculo	Celetista	18	22,79
	Estatutário	35	44,30
	Contrato por tempo determinado	26	32,91
Outro vínculo empregatício	Não	39	49,37
	Sim	40	50,63
Experiência em cargo gerencial	Não	49	62,02
	Sim	30	37,98

Fonte: Elaboração própria.

**Tabela 2:** Caracterização dos enfermeiros das unidades de APH (móvel e fixo) quanto às variáveis: idade, tempo de formação e tempo na instituição – Belo Horizonte, MG, Brasil, 2023 (N = 79).

Variáveis	Média	Desvio- padrão	Mínimo (anos)	Máximo (anos)
Idade	41,10	8,12	24	68
Tempo de formação	13,66	7,32	1	34
Tempo na instituição	5,95	1,32	6 meses	26

Fonte: Elaboração própria.

Nota-se pela Tabela 1 que há um número expressivo de enfermeiras (n = 61; 77,22%). A maior parte dos profissionais que participaram da pesquisa sinaliza possuir especialização (n = 70; 88,61%), ter vínculo empregatício estatutário (n = 35; 44,30%), possuir outro vínculo empregatício (n = 40; 50,63%) e não ter experiência em cargo gerencial (n = 49; 62,02%). Já na Tabela 2 observa-se que em média: a idade dos profissionais foi de 41,1 anos de idade (DP = 8,12); o tempo de formação foi de 13,66 anos (DP = 7,32) e; o tempo na instituição foi de 5,95 anos (DP = 1,32).

A Tabela 3 corresponde às análises de correlação entre os domínios dos instrumentos aplicados no presente estudo. Embora tenha se evidenciado uma correlação baixa entre o escore total do QUAPEEL e do CET-II e entre os domínios do instrumento, nota-se que a Liderança *Coaching* influencia o empoderamento dos enfermeiros atuantes no contexto do APH, fato esse constatado a partir de uma quantidade significativa de correlações ente o escore total do QUAPEEL com os domínios do instrumento que mensura o empoderamento estrutural (informação, suporte, recursos e poder formal).

**Tabela 3:** Correlação da prática da Liderança Coaching e do empoderamento estrutural dos enfermeiros das unidades de APH – Belo Horizonte, MG, Brasil, 2023 (N = 79).

Domínios	Oportunidade	Informação	Suporte	Recursos	Poder formal	Poder informal	Escore total do CET-II
Comunicação	0,317*	**0,258**	**0,326**	**0,477**	**0,353**	**0,262**	**0,467**
	0,004**	**0,022**	**0,003**	**<0,001**	**0,001**	**0,020**	**<0,001**
Dar e receber feedback	**0,227**	**0,221**	**0,278**	**0,366**	**0,360**	0,062	**0,366**
	**0,045**	**0,050**	**0,013**	**<0,001**	**0,001**	0,584	**<0,001**
Dar e exercer influência	0,113	0,201	0,147	0,165	**0,246**	–0,129	0,189
	0,321	0,076	0,196	0,145	**0,029**	0,258	0,096
Apoiar a equipe para alcance de resultados	0,122	0,202	**0,246**	0,205	**0,314**	0,010	**0,272**
	0,286	0,074	**0,029**	0,069	**0,005**	0,928	**0,015**
**Escore total do QUAPEEL**	0,216	**0,266**	**0,298**	**0,340**	**0,386**	0,037	**0,376**
	0,056	**0,018**	**0,008**	**0,002**	**<0,001**	0,747	**<0,001**

Fonte: Elaboração própria;

*Correlação de Pearson;

**p-valor.

Em uma análise mais pormenorizada dos domínios do QUAPEEL, a “comunicação” foi que apresentou correlação com todos os domínios do CET-II, a incluir o Escore total do instrumento, o que demonstra a relevância dessa competência para a liderança do enfermeiro nesse cenário. Em sequência, “dar e receber feedback” foi o segundo constructo a influenciar o empoderamento estrutural, não apresentando correlação entre esse domínio do QUAPEEL e poder informal presente no CET-II somente. “Dar e exercer influência” foi o domínio com menor correlação com os constructos do instrumento de empoderamento estrutural, uma vez que apresentou correlação apenas com o poder formal, denotando assim uma competência a ser aprimorada pelos profissionais.

## DISCUSSÃO

Em relação à caracterização dos participantes, a maior parte dos profissionais foi composta por enfermeiras que possuíam especialização, além de ter as médias de: 41,1 anos de idade; 13,66 anos de tempo de formação e 5,95 anos de tempo na instituição. Esses achados se aproximam com os três estudos nacionais que aplicaram o QUAPEEL no contexto do APH^(4)^ e o CET-II no contexto da urgência/emergência hospitalar^(18,19)^.

Já o resultado relacionado a possuir outro vínculo empregatício foi divergente ao encontrado em um estudo desenvolvido em um SAMU no Distrito Federal, cuja porcentagem foi maior para pessoas sem um segundo vínculo empregatício^(20)^. Não possuir experiência em cargo gerencial e ter vínculo empregatício estatutário resultou em um percentual próximo ao de dois estudos no contexto da urgência e emergência nacional, sendo um no estado de Goiás^(9)^ e o outro na região metropolitana de Belo Horizonte^(18)^.

Identificou-se no presente estudo que o maior número de enfermeiros não apresentava experiência em cargos gerenciais. Diante disso, salienta-se que o enfermeiro emergencista, especialmente, quando já possui experiências no âmbito da gestão, consegue responder e se adaptar efetivamente aos desafios atuais e futuros das práticas do gerenciamento do cuidado^(6)^.

Na presente pesquisa, evidenciou-se que o escore total do QUAPEEL obteve correlação fraca positiva com praticamente todos os constructos e com o escore total do CET-II (R = 0,376; *p*-valor = <0,001). Salienta-se que, dessa maneira, o papel dos estilos e modelos liderança do enfermeiro favorecem o empoderamento desses profissionais.

Acerca dessa relação, dois artigos recentes^(20,21)^, no contexto da urgência e emergência, expressam o potencial dessa competência, nos diferentes modelos e estilos, sobre os elementos do empoderamento estrutural. O estudo realizado na Jordânia observou que as práticas de liderança clínica mostraram ter uma relação positiva significativa com o empoderamento estrutural (r = 0,65; P < 0,01)^(21)^; por sua vez, enfermeiros do departamento de emergência do Nepal, que exerciam a liderança colaborativa para empoderar suas equipes, obtiveram maior adesão e melhores respostas aos treinamentos sobre atendimento inicial ao trauma^(22)^.

As experiências dos liderados na enfermagem podem ser afetadas diretamente pela forma como o enfermeiro exercita sua liderança e estabelece condições de trabalho favoráveis para o desenvolvimento da sua equipe. Sob essa ótica, revisão sistemática brasileira^(23)^ apresenta evidências de como a liderança impacta no empoderamento estrutural, consequentemente influenciando na satisfação no trabalho, engajamento e aumento da qualidade da assistência prestada^(23)^.

Embora o escore total do QUAPEEL não tenha apresentado correlação com o domínio “Oportunidade” do CET-II, revisão integrativa acerca do empoderamento no contexto da urgência e emergência sinaliza que, para o alcance da autonomia no trabalho e para melhores níveis de autoeficácia pelas oportunidades, é fundamental que os enfermeiros estejam alicerçados em modelos positivos de liderança como a ressonante e a clínica^(13)^. Ademais, o aumento de oportunidade desse profissional na RUE pode ser percebido pela Enfermagem de Práticas Avançadas, o que representa o crescimento e o papel ampliado do enfermeiro no APH brasileiro^(24)^.

Evidenciou-se a partir da Tabela 3 como a “comunicação” na autopercepção do enfermeiro é um componente expressivo no modelo de Liderança *Coaching,* posto que influencia significativamente no seu empoderamento. A comunicação dos enfermeiros líderes é crucial para construir boas relações e empoderar os membros da equipe. Para tanto, ela deverá alicerçar-se nos pilares da comunicação: um diálogo de apoio, em vez de impositivo; o compartilhamento das visões e valores do líder e; a comunicação e o suporte das práticas baseadas em evidências^(25)^.

Sob a lógica do liderado, dois trabalhos^(4,9)^ que analisaram a percepção de técnicos de enfermagem sobre o exercício da Liderança *Coaching* pelos seus coordenadores no contexto APH móvel, a “comunicação” foi o segundo domínio com a maior média, correlacionando-se com o menor tempo de formação^(4)^ e com a satisfação no trabalho^(9)^. A comunicação na Liderança *Coaching*, segundo revisão integrativa recente^(12)^, tem um impacto significativo no suporte individual dos profissionais de enfermagem liderados, o que reflete no desenvolvimento de novas competências desses profissionais que os possibilite enxergar novos caminhos para o trabalho em equipe^(12)^.

“Dar e receber o feedback” do instrumento QUAPEEL apenas não se correlacionou com o poder informal, domínio do CET, o que demonstra a relevância desse instrumento no processo do empoderamento. Nesse sentido, dois estudos convergem para esse resultado^(12,26)^. O primeiro analisa a liderança dos enfermeiros de prática avançada e revelou que essa competência, quando baseada no processo *coaching*, promove o empoderamento e a melhoria de seu desempenho do grupo, motivo pelo qual fica evidente a necessidade de os enfermeiros valorizarem os mecanismos de feedback^(26)^.

Já o segundo trabalho, uma revisão integrativa^(12)^, ressaltou o feedback na liderança *coaching* como um facilitador para o empoderamento dos enfermeiros. Com isso, as organizações de saúde e seus profissionais podem ter um alto desempenho e atender aos padrões e aos protocolos institucionais^(12)^. É necessário que os enfermeiros estejam capacitados e despendam o tempo adequado/apropriado para feedback, especialmente quando for abordar aspectos como o erro. Destarte, o líder deve ser capaz de ofertar o apoio necessário e promover a cultura da não punição, além de analisar as falhas de forma sistemática^(25)^.

O poder formal, conforme o referencial de empoderamento estrutural, compreende os aspectos relacionados à posição hierárquica que um indivíduo possui dentro de uma organização^(10)^. Esse constructo do instrumento CET-II foi o único que se correlacionou com a dimensão da Liderança *Coaching* “dar e exercer influência”, conforme observado na Tabela 3.

A influência do enfermeiro é uma habilidade a ser estimulada nos profissionais das unidades do APH estudadas, tendo em vista os resultados das correlações. Esse achado evidencia que a capacidade de inspirar e motivar equipes, aliada à competência de comunicação eficaz e tomada de decisão rápida são fundamentais para garantir a qualidade da assistência e a segurança do paciente nesse contexto dinâmico e desafiador que compreende a urgência e a emergência^(6,24)^.

Embora a dimensão “Apoiar a equipe para o alcance dos resultados” tenha apresentado menor correlação com os domínios do CET-II, estudo recente^(5)^ da rede de urgência e emergência de um município no interior paulista apontou a necessidade de o enfermeiro líder na construção de credibilidade para obter o apoio de todos os níveis de atuação, assim como em todos os cenários. Considerando-se que o SAMU e as UPA se inserem no contexto da REU, para empoderar-se, o enfermeiro precisa, no exercício de sua liderança, compreender as políticas internas e externas, analisar criticamente as demandas que surgem e reconhecer o espaço e as oportunidades de liderança a terceiros^(5)^.

A limitação da pesquisa esteve relacionada, principalmente, ao número de profissionais que não foram encontrados em seus plantões para serem convidados a participarem da pesquisa. Mesmo assim, essa limitação não impactou fortemente nas análises estatísticas e tampouco minimizou a relevância e o caráter inovador da pesquisa ao apresentar uma investigação acerca da liderança dos enfermeiros não somente o contexto do APH móvel, como também as Unidades de Pronto Atendimento Fixo, na qualidade das UPA. Além disso, a realização do estudo possibilitou ainda analisar a correlação de duas variáveis que têm uma relevância para a prática profissional do enfermeiro da urgência e emergência.

As contribuições da presente pesquisa demonstram a importância da prática da Liderança *Coaching* no empoderamento dos enfermeiros atuantes no contexto do APH móvel e fixo, com destaque para a correlação positiva entre a “comunicação” e todos os domínios do empoderamento estrutural. Esse achado tem implicações diretas na prática profissional, pois sugere que o desenvolvimento de habilidades comunicativas pelos enfermeiros pode fortalecer sua capacidade de influenciar e liderar a equipe de maneira mais eficaz, melhorando a organização e os resultados do cuidado. Além disso, a relação obtida entre “dar e receber feedback” e o empoderamento estrutural reforça a necessidade de práticas de avaliação e de apoio contínuo entre os profissionais.

## CONCLUSÃO

Os achados do presente artigo demonstram que a Liderança *Coaching* exercida pelos enfermeiros do APH móvel e fixo apresenta uma correlação positiva com o empoderamento estrutural. Esse aspecto foi observado pela correlação entre os escores totais dos instrumentos utilizados na pesquisa, bem como pelas inúmeras correlações entre os domínios do QUAPEEL e CET-II. Dessa maneira, tais descobertas orientam estratégias de capacitação focadas em elementos e competências tais como a comunicação e o feedback, o que repercute positivamente na qualidade do atendimento, na satisfação dos pacientes e no desenvolvimento profissional contínuo dos enfermeiros no APH.

## Data Availability

Todo o conjunto de dados que dá suporte aos resultados deste estudo foi publicado no próprio artigo.

# Translation: Liderazgo, *coaching* y empoderamiento estructural de los enfermeros del servicio de atención prehospitalaria móvil y fija

**DOI:** 10.1590/1980-220X-REEUSP-2024-0450es

**Authors:** Letícia Santiago Utsch, Fernanda Oliveira Santos, Katharina Júlia Lacerda Oliveira, Hercules de Oliveira Carmo, André Almeida de Moura

**Affiliations:** 1 Universidade Federal de Minas Gerais, Escola de Enfermagem, Belo Horizonte, MG, Brasil.; 2 Universidade de São Paulo, Escola de Enfermagem, Departamento de Orientação Profissional, São Paulo, SP, Brasil.

**Keywords:** Liderazgo, Empoderamiento, Enfermería de Urgencia, Enfermeras y Enfermeros, Servicios Médicos de Urgencia

## Abstract

**Objetivo:**

Analizar la correlación entre el liderazgo *coaching* ejercido por los enfermeros de la Atención Prehospitalaria (APH) móvil y fija con el empoderamiento estructural de estos profesionales.

**Método:**

Estudio analítico y descriptivo con 79 enfermeros de la atención prehospitalaria móvil y fija. Se utilizaron los siguientes cuestionarios: Caracterización de los sujetos; Cuestionario de Autopercepción del Enfermero en el Ejercicio del Liderazgo; y Cuestionario de Condiciones de Eficacia en el Trabajo II (CET-II). Se emplearon el análisis descriptivo y el análisis inferencial con el uso de la prueba de Spearman para determinar las correlaciones entre las dimensiones del instrumento, adoptando un nivel de significación del 5%.

**Resultados:**

El liderazgo *coaching* influye positivamente en el empoderamiento de los enfermeros que trabajan en el contexto de la APH, como lo demuestra la débil correlación entre las puntuaciones totales de los instrumentos aplicados en la investigación (R = 0,376; *p*-valor = <0,001). La “comunicación” se correlacionó con todos los dominios del CET-II, incluida la “puntuación total” del instrumento.

**Conclusión:**

Es importante que las organizaciones de APH móviles y fijas inviertan en el liderazgo de sus enfermeros, a partir del uso del modelo de Liderazgo Coaching, teniendo en cuenta especialmente que tiene una influencia positiva en el empoderamiento del enfermero.

## INTRODUCCIÓN

La actuación del enfermero en el servicio de urgencias y emergencias es de gran relevancia, ya que este profesional está en contacto directo con el paciente desde el primer momento (en el lugar del suceso) a través de la Atención Prehospitalaria (APH) móvil, en calidad de Servicio de Atención Móvil de Urgencias (SAMU), pasando por la atención prehospitalaria fija que comprenden las Unidades de Atención de Urgencias (UPA), hasta el hospital que acoge y presta atención intrahospitalaria a las necesidades agudas de salud. Todos estos escenarios de atención exigen al enfermero actitudes, habilidades, conocimientos técnico-científicos, además del cumplimiento de aspectos éticos relacionados con la urgencia y la emergencia^(1)^.

En Brasil, la Red de Atención a Urgencias y Emergencias (RUE) tiene como objetivo desarrollar acciones de promoción, prevención y vigilancia de la salud, así como la atención y el cuidado de los usuarios en condiciones agudas. Ante este escenario, el proceso de trabajo en urgencias exige un dinamismo significativo por parte de los profesionales en respuesta a las altas demandas, los eventos inesperados, la capacitación profesional constante, la sinergia y la intercomunicación en el equipo, tanto antes como dentro del hospital, para promover una atención segura y de calidad^(2)^.

Así, teniendo en cuenta este dinamismo, los enfermeros desempeñan un papel indispensable en esta atención, principalmente en el ejercicio del liderazgo en su práctica profesional. Es a través de esta competencia que dicho profesional logra mejores resultados para los pacientes y los equipos y promueve el diálogo con los demás servicios y sectores de la salud^(3,4)^. Por lo tanto, se percibe que el liderazgo del enfermero en este contexto busca favorecer las dimensiones de su proceso de trabajo^(5)^.

Un editorial reciente^(6)^ de una revista internacional de enfermería de urgencias presenta una reflexión sobre el liderazgo como competencia fundamental para la práctica de la enfermería en el sector de urgencias y subraya que, para su movilización efectiva, este profesional debe actualizarse constantemente y asociar al liderazgo el uso de otras competencias y herramientas de gestión. Por lo tanto, para que el enfermero pueda ejercer esta competencia, dicho profesional deberá superar algunos retos, como la sobrecarga de trabajo y la demanda, valiéndose de estrategias para superarlos. Para ello, el texto señala que, además del liderazgo, el enfermero necesita dominar una amplia gama de herramientas, como el pensamiento estratégico, la resolución de problemas, la inteligencia emocional, la colaboración, la formación de equipos y la toma de decisiones.

Corroborando este editorial, un estudio etnográfico con enfermeros del servicio prehospitalario sueco afirma que, a partir de su responsabilidad y liderazgo, el enfermero tiene un papel fundamental en la elucidación de los valores de sus subordinados, incentivándolos a desarrollar sus propias cualidades y promoviendo, en consecuencia, un ambiente de práctica favorable en el equipo de enfermería^(7)^.

Ante la magnitud del liderazgo para la actuación del enfermero en urgencias, se señala el modelo de Liderazgo Coaching como referencia para el ejercicio de esta competencia por parte de los enfermeros. Conceptualmente, el Liderazgo Coaching se basa en el proceso de coaching, que consiste en una competencia a partir de la cual el líder busca influir en el equipo para alcanzar los objetivos, al mismo tiempo que posibilita el desarrollo de sus subordinados. Para ello, este modelo se basa en cuatro dimensiones del proceso de coaching, a saber: “Comunicación”, “Dar y recibir feedback”, “Dar poder y ejercer influencia” y “Apoyo al equipo”^(4,8)^.

Basándose en este modelo de liderazgo en la APH, dos artículos brasileños^(4,9)^, desarrollados en el estado de Goiás, revelaron que los enfermeros, al ejercer el Liderazgo *Coaching*, promueven transformaciones positivas en su espacio de trabajo, como la satisfacción de sus subordinados y, de la misma manera, permiten la prestación de cuidados de calidad y seguridad a la población atendida por este servicio. De este modo, se observa que el liderazgo en la APH promueve una optimización del entorno y la organización del proceso de trabajo de la enfermería, con el fin de garantizar que los enfermeros tengan la capacidad de desarrollar sus prácticas en un ámbito más completo y, así, ofrecer una atención de alta calidad.

Por lo tanto, de acuerdo con este tipo de liderazgo, un buen líder tiene la capacidad de: construir relaciones de confianza y entornos de trabajo más fructíferos; estimular la autonomía de sus profesionales; compartir el poder y, en consecuencia, obtener una mayor excelencia en la atención y la seguridad del paciente. La competencia del liderazgo en enfermería tiene una gran influencia en la práctica profesional, especialmente cuando se analiza el empoderamiento estructural. Los enfermeros líderes menos empoderados estructuralmente tienen más probabilidades de frustrarse al intentar mejorar su práctica profesional e innovar en la atención al paciente^(10,11)^.

Una revisión integradora^(12)^, publicada en una importante revista internacional, evidencia que el Liderazgo *Coaching* sigue siendo un área emergente de la investigación en enfermería. Además, los autores del trabajo señalan que el desarrollo de investigaciones basadas en el marco de referencia del Liderazgo *Coaching* puede resultar en subsidios para que los servicios de salud inviertan en las dimensiones del proceso de *coaching*, con el fin de construir el empoderamiento de los enfermeros y, por consiguiente, proporcionar las competencias necesarias para que estos profesionales desarrollen su trabajo al más alto nivel.

Los hallazgos de nuevos trabajos sobre el tema se establecen como una oportunidad para explorar más a fondo los beneficios, los retos y las formas en que el Liderazgo Coaching puede implementarse para apoyar a los enfermeros en su desarrollo y su práctica. Así, al analizar el contexto de la APH fija y móvil, aún son incipientes las investigaciones que exploran las interfaces de este modelo de liderazgo establecido por los enfermeros con otras variables^(4,9)^, como el empoderamiento^(13)^. Para el presente, se adoptó el empoderamiento estructural de Kanter, que entiende el poder como la capacidad de realizar acciones y movilizar y emplear los recursos necesarios para alcanzar los objetivos organizacionales^(10)^.

Como hipótesis, el presente artículo buscó comprobar si la autopercepción de los enfermeros sobre el Liderazgo Coaching se correlaciona con el empoderamiento estructural, teniendo en cuenta el contexto de la APH móvil y fija. En vista de lo anterior, el presente artículo buscó analizar la correlación entre el liderazgo coaching ejercido por los enfermeros de la APH móvil y fija con el empoderamiento estructural de estos profesionales.

## MÉTODO

### Tipo de Estudio

Se trata de un estudio descriptivo y analítico, con el objetivo de examinar la relación entre las variables del Liderazgo *Coaching* y el empoderamiento estructural de los enfermeros que trabajan en la APH móvil y fija. Además, la presente investigación siguió las recomendaciones del Strengthening the Reporting of Observational Studies in Epidemiology (STROBE)^(14)^ para respaldar la comunicación de los hallazgos y las etapas recorridas.

### Lugar del Estudio

El escenario del estudio correspondió a la RUE presente en una capital metropolitana de la región sureste del país. Cabe destacar, asimismo, que la organización de los servicios de APH (móvil y fija) en este municipio está constituida por la central del SAMU, seis bases descentralizadas, además de las nueve UPA. Teniendo en cuenta estas últimas unidades, se enviaron correos electrónicos a las coordinaciones generales y de enfermería y tres de las nueve UPA no respondieron a los correos electrónicos para la Recolección de datos.

### Sujetos de la Investigación

En el momento de realizar la investigación, el total de enfermeros de las unidades (SAMU y UPA) era de 258 profesionales, tanto en el ámbito asistencial como en el de la gestión. Se estableció la muestra por conveniencia, es decir, no probabilística, con los enfermeros que realizaban acciones asistenciales o actividades de gestión en las unidades analizadas, dentro de los criterios de inclusión establecidos.

Se incluyeron en la investigación los enfermeros que llevaban al menos seis meses en el cargo, con el fin de garantizar que el trabajador ya se sintiera adaptado y capaz de evaluar la práctica del liderazgo coaching y el empoderamiento estructural (39 profesionales no cumplían este criterio). En cuanto a los criterios de exclusión, no participaron en la investigación los profesionales de la categoría mencionada anteriormente que estaban disfrutando de vacaciones o de un permiso especial (n = 34), o que estaban ausentes por motivos de salud (n = 10). Cabe señalar que 21 profesionales se negaron a participar en la investigación, lo que supuso un total de 154 enfermeros elegibles para participar en la investigación.

A partir de los 154 profesionales, se realizó el cálculo de la muestra, adoptando como parámetros un error muestral del 8%, un nivel de confianza del 95% y una distribución de la población más homogénea, lo que dio como resultado un número mínimo de 61 enfermeros como representación de la población. El número de encuestados (79 enfermeros) fue superior al establecido por el cálculo de la muestra. El cálculo no se estratificó para las unidades (APH móvil y fija), ya que se buscaba un análisis global del contexto de la APH. Cabe destacar que se consideraron como pérdida de la muestra: los profesionales que, tras dos intentos, no fueron localizados en sus respectivos turnos y aquellos que no devolvieron los cuestionarios cumplimentados o los devolvieron en blanco.

### Instrumentos Utilizados en la Recolección de Datos

Se aplicaron tres cuestionarios, a saber: la caracterización de los sujetos, que contiene datos sociodemográficos y relacionados con la profesión; el Cuestionario de Autopercepción del Enfermero en el Ejercicio del Liderazgo (Questionário de Autopercepção do Enfermeiro no Exercício da Liderança – QUAPEEL por su sigla en portugués)^(8)^ y; el cuestionario, validado transculturalmente para Brasil, de Condiciones de Eficacia en el Trabajo II (CET-II), que analiza el empoderamiento estructural^(15)^.

Los datos sociodemográficos y relacionados con la profesión fueron: sexo (masculino y femenino); formación (especialización, *Master in Business Administration* MBA, maestría, doctorado; curso complementario – soporte vital básico, etc.); tipo de vínculo laboral (estatutario, celetista, contrato por tiempo determinado y otros); tener otro vínculo laboral; haber ocupado un cargo directivo; edad (cálculo de la diferencia entre las fechas de Recolección y de nacimiento); tiempo de formación (diferencia entre las fechas de Recolección y de finalización de la licenciatura) y; tiempo de actuación en la unidad (tiempo transcurrido entre las fechas de Recolección y de admisión en la unidad).

El QUAPEEL, que fue elaborado y validado en Brasil, cuenta con preguntas estructuradas y compuestas por tres partes: la primera corresponde a los datos sociodemográficos de los sujetos; la segunda consiste en preguntas relacionadas con los conocimientos de los sujetos sobre el tema del liderazgo, mientras que la tercera presenta preguntas relacionadas con las habilidades y actitudes ejercidas por los líderes en la práctica del Liderazgo *Coaching* a través de la autopercepción del enfermero^(8)^. Cabe destacar que para la presente investigación se utilizó únicamente la tercera parte del cuestionario.

Este cuestionario consta de 20 ítems, subdivididos en cuatro ámbitos: Comunicación; Dar y recibir feedback; Dar y ejercer influencia; Apoyar al equipo para alcanzar resultados. El cuestionario utiliza una escala Likert, con la siguiente configuración para cada ítem: Nunca (1); Rara vez (2); No siempre (3); Casi siempre (4) y; Siempre (5). La opción “No aplica” (NA), incluida en la escala Likert, corresponde al caso en que el profesional cree que no desarrolla dicha acción; para ello, se le ha asignado el valor cero (0). Por lo tanto, la variación de la puntuación general del instrumento está entre los valores de 0 a 100, es decir, los valores que se acercan a 0 corresponden a una menor percepción de la práctica del Liderazgo *Coaching*, mientras que, en las puntuaciones cercanas a 100, mayor es la percepción de la práctica de este tipo de liderazgo^(8)^.

El Cuestionario de Condiciones de Eficacia en el Trabajo II (CET-II), versión adaptada transculturalmente, se utilizó con el objetivo de analizar el empoderamiento estructural en el entorno de trabajo de los enfermeros de la APH^(15)^. El instrumento original fue concebido y desarrollado por investigadores canadienses, teniendo como referencia teórica el empoderamiento de Kanter^(16)^.

En Brasil, la adaptación y validación transcultural de este instrumento fue realizada por un grupo de investigadores de Paraná, con el fin de evaluar el trabajo de los enfermeros en diferentes escenarios, en relación con las condiciones de empoderamiento estructural. Cabe reiterar que el presente cuestionario es ampliamente utilizado por los investigadores de la área y, en el proceso de validación transcultural, obtuvo un alfa de Cronbach de 0,88, lo que representa una alta consistencia interna entre las respuestas^(15)^.

El CET-II se estructura en 20 ítems, divididos en seis dimensiones: Oportunidad; Información; Apoyo; Recursos; Poder formal y Poder informal. Existe además un constructo que engloba una perspectiva más amplia del empoderamiento, con dos ítems para analizar el empoderamiento global, pero que no se han aplicado en la presente investigación, ya que solo se analizan las seis dimensiones mencionadas anteriormente^(16)^.

La medición del cuestionario CET-II se realiza mediante una escala tipo Likert, cuya puntuación varía de 1 (ninguno/ninguna) a 5 (mucho/mucha), mientras que los valores entre 2 y 4 representan valores intermedios. Los elementos de cada uno de los componentes se suman y, a continuación, se calcula una media con el fin de proporcionar una puntuación que varía entre 1 y 5 para cada dimensión, como se ha mencionado anteriormente. La suma de las dimensiones puede variar de 6 a 30, siendo que los valores entre 6 y 13 significan bajos niveles de empoderamiento; entre 14 y 22 significan niveles moderados de empoderamiento y entre 23 y 30 significan altos niveles de empoderamiento^(15,16)^.

### Recolección y Análisis de Datos

Un grupo formado por un profesor investigador y cuatro alumnas se encargó de entregar los cuestionarios a los sujetos que aceptaron participar en la investigación. Para ello, se contactó previamente con los coordinadores administrativos y de enfermería de las unidades en cuestión para que se entregaran los cuestionarios a los enfermeros personalmente, así como para programar la recogida de los instrumentos.

Los datos recopilados a través de los cuestionarios se introdujeron dos veces en hojas de cálculo y se compararon, asegurándose así que no hubiera errores de digitación, y posteriormente se realizaron análisis estadísticos descriptivos y de correlación entre las variables. Para ello, se utilizó el programa estadístico R versión 4.3.3 para el tratamiento de los datos. Las variables cualitativas se resumieron en frecuencias simples y relativas utilizando porcentajes. Las variables cuantitativas se organizaron por medias, medianas, desviación estándar (DE), primer y tercer cuartil y valores mínimos y máximos.

Inicialmente, se utilizó la prueba de normalidad de Shapiro-Wilk para evaluar la distribución de las variables. Para el análisis de la correlación entre la autopercepción del Liderazgo *Coaching* y el empoderamiento estructural se aplicó la correlación de Pearson, que puede ser positiva o negativa. Se utilizó un nivel de significación del 5% para todas las pruebas estadísticas (α = 0,05). Además, las correlaciones se interpretaron/evaluaron según el tamaño de la correlación, positiva o negativa, pudiendo presentar: correlación muy alta (valores superiores a 0,9 hasta 1,0; o superiores a –0,9 hasta –1,0); correlación alta (valores superiores a 0,7 hasta 0,9; o superiores a –0,7 hasta –0,9); correlación moderada (valores superiores a 0,5 hasta 0,7 o superiores a –0,5 hasta –0,7); correlación baja (valores superiores a 0,3 hasta 0,5 o valores superiores a –0,3 hasta –0,5) y; correlación insignificante (valores superiores a 0,0 hasta 0,3 o superiores a 0,0 hasta –0,3)^(17)^.

### Aspectos Éticos de la Investigación

La investigación fue evaluada y aprobada por los Comités de Ética en Investigación de la Universidad Federal de Minas Gerais, con el número 5.904. 124 (el 20 de febrero de 2023), y por la Secretaría Municipal de Salud de Belo Horizonte, con el número 6.063.014 (el 16 de mayo de 2023), a fin de cumplir con las disposiciones de la Resolución n.º 466/2012 del Consejo Nacional de Salud. Además, todos los participantes recibieron orientación sobre la investigación y firmaron el Término de Consentimiento Libre e Informado antes de responder los cuestionarios.

## RESULTADOS

El número de enfermeros que participaron en la investigación durante el período de Recolección de datos fue de 79 profesionales, lo que correspondió al 48,17% de los participantes elegibles. En cuanto a las pérdidas de la muestra: 22 enfermeros no fueron localizados en sus respectivos turnos, tras dos intentos infructuosos, y 53 profesionales no completaron o devolvieron los cuestionarios en blanco. Los datos correspondientes a la caracterización de los enfermeros en cuanto a las variables sociodemográficas o relacionadas con la profesión se presentan en las Tablas 1 y 2.

**Tabla 1:** Caracterización de los enfermeros de las unidades de APH (móviles y fijas) en cuanto a las variables: sexo, formación (especialización, MBA, maestría, doctorado, curso complementario), tipo de vínculo, tener otro vínculo laboral y experiencia en puestos gerenciales – Belo Horizonte, MG, Brasil, 2023 (N = 79).

Variables	Descripción	N	Porcentaje (%)
Sexo	Femenino	61	77,21
	Masculino	18	22,79
Especialización	No	9	11,39
	Si	70	88,61
MBA	No	74	93,67
	Si	5	6,33
Maestría	No	69	87,34
	Si	10	12,66
Doctorado	No	78	98,73
	Si	1	1,27
Curso complementario	No	42	53,16
	Si	37	46,84
Tipo de vínculo	Contrato regulado	18	22,79
	Estatutario	35	44,30
	Contrato por tiempo determinado	26	32,91
Otro vínculo de empleo	No	39	49,37
	Si	40	50,63
Experiencia en cargo gerencial	No	49	62,02
	Si	30	37,98

Fuente: Elaborado por los autores.

**Tabla 2:** Caracterización de los enfermeros de las unidades de APH (móviles y fijas) en cuanto a las variables: edad, tiempo de formación y tiempo en la institución – Belo Horizonte, MG, Brasil, 2023 (N = 79).

Variables	Media	Desviación estándar	Mínimo (años)	Máximo (años)
Edad	41,10	8,12	24	68
Tiempo de formación	13,66	7,32	1	34
Tiempo en la institución	5,95	1,32	6 meses	26

Fuente: Elaborado por los autores.

En la Tabla 1 se observa que hay un número significativo de enfermeros (n = 61; 77,22%). La mayoría de los profesionales que participaron en la encuesta indican que tienen una especialización (n = 70; 88,61%), un vínculo laboral público estatutario (n = 35; 44,30%), otro tipo de vínculo laboral (n = 40; 50,63%) y no tener experiencia en puestos directivos (n = 49; 62,02%). En la Tabla 2 se observa que, en promedio: la edad de los profesionales era de 41,1 años (DE = 8,12); el tiempo de formación era de 13,66 años (DE = 7,32) y; el tiempo en la institución era de 5,95 años (DE = 1,32).

La Tabla 3 corresponde a los análisis de correlación entre los dominios de los instrumentos aplicados en el presente estudio. Aunque se evidenció una baja correlación entre la puntuación total del QUAPEEL y el CET-II y entre los dominios del instrumento, se observa que el Liderazgo *Coaching* influye en el empoderamiento de los enfermeros que trabajan en el contexto de la APH, hecho que se constata a partir de una cantidad significativa de correlaciones entre la puntuación total del QUAPEEL y los dominios del instrumento que mide el empoderamiento estructural (información, apoyo, recursos y poder formal).

**Tabla 3:** Correlación entre la práctica del liderazgo coaching y el empoderamiento estructural de los enfermeros de las unidades de APH – Belo Horizonte, MG, Brasil, 2023 (N = 79).

Dominios	Oportunidad	Información	Apoyo	Recursos	Poder formal	Poder informal	Puntuación total do CET-II
Comunicación	**0,317** *	**0,258**	**0,326**	**0,477**	**0,353**	**0,262**	**0,467**
	0,004**	**0,022**	**0,003**	**<0,001**	**0,001**	**0,020**	**<0,001**
Dar y recibir feedback	**0,227**	**0,221**	**0,278**	**0,366**	**0,360**	0,062	**0,366**
	**0,045**	**0,050**	**0,013**	**<0,001**	**0,001**	0,584	**<0,001**
Dar y ejercer influencia	0,113	0,201	0,147	0,165	**0,246**	–0,129	0,189
	0,321	0,076	0,196	0,145	**0,029**	0,258	0,096
Apoyar al equipo para alcance de resultados	0,122	0,202	**0,246**	0,205	**0,314**	0,010	**0,272**
	0,286	0,074	**0,029**	0,069	**0,005**	0,928	**0,015**
**Escore total del QUAPEEL**	0,216	**0,266**	**0,298**	**0,340**	**0,386**	0,037	**0,376**
	0,056	**0,018**	**0,008**	**0,002**	**<0,001**	0,747	**<0,001**

Fuente: Elaborado por los autores;

*Correlación de Pearson;

**p-valor.

En un análisis más detallado de los dominios del QUAPEEL, la “comunicación” fue la que presentó correlación con todos los dominios del CET-II, incluida la puntuación total del instrumento, lo que demuestra la relevancia de esta competencia para el liderazgo del enfermero en este escenario. A continuación, “dar y recibir feedback” fue el segundo constructo que influyó en el empoderamiento estructural, sin presentar correlación entre este dominio del QUAPEEL y el poder informal presente solo en el CET-II. “Dar y ejercer influencia” fue el dominio con menor correlación con los constructos del instrumento de empoderamiento estructural, ya que solo presentó correlación con el poder formal, lo que denota una competencia que debe ser mejorada por los profesionales.

## DISCUSIÓN

En cuanto a la caracterización de los participantes, la mayoría de los profesionales eran enfermeras con especialización, con una media de 41,1 años de edad, 13,66 años de formación y 5,95 años de antigüedad en la institución.

Estos resultados se acercan a los de tres estudios nacionales que aplicaron el QUAPEEL en el contexto de la APH^(4)^ y el CET-II en el contexto de urgencias/emergencias hospitalarias^(18,19)^.

Por otro lado, el resultado relacionado con tener otro vínculo laboral fue diferente al encontrado en un estudio realizado en un SAMU en el Distrito Federal, donde el porcentaje fue mayor para las personas sin un segundo vínculo laboral^(20)^. No tener experiencia en puestos gerenciales y tener un vínculo laboral estatutario dio como resultado un porcentaje similar al de dos estudios en el contexto de urgencias y emergencias nacionales, uno en el estado de Goiás^(9)^ y otro en la región metropolitana de Belo Horizonte^(18)^.

En el presente estudio se identificó que la mayoría de los enfermeros no tenían experiencia en puestos gerenciales. Ante esto, cabe destacar que el enfermero de urgencias, especialmente cuando ya tiene experiencia en el ámbito de la gestión, es capaz de responder y adaptarse eficazmente a los retos actuales y futuros de las prácticas de gestión de la atención^(6)^.

Nuestra investigación evidenció que la puntuación total del QUAPEEL obtuvo una correlación positiva débil con prácticamente todos los constructos y con la puntuación total del CET-II (R = 0,376; valor p = <0,001). Cabe destacar que, de esta manera, el papel de los estilos y modelos de liderazgo de los enfermeros favorece el empoderamiento de estos profesionales.

En relación con esta relación, dos artículos recientes^(20,21)^, en el contexto de la urgencia y la emergencia, expresan el potencial de esta competencia, en los diferentes modelos y estilos, sobre los elementos del empoderamiento estructural. El estudio realizado en Jordania observó que las prácticas de liderazgo clínico mostraron tener una relación positiva significativa con el empoderamiento estructural (r = 0,65; P < 0,01)^(21)^; a su vez, los enfermeros del departamento de urgencias de Nepal, que ejercían el liderazgo colaborativo para empoderar a sus equipos, obtuvieron una mayor adhesión y mejores respuestas a las capacitaciones sobre atención inicial al trauma^(22)^.

Las experiencias de los liderados en enfermería pueden verse afectadas directamente por la forma en que el enfermero ejerce su liderazgo y establece condiciones de trabajo favorables para el desarrollo de su equipo. Desde esta perspectiva, una revisión sistemática brasileña^(23)^ presenta evidencias de cómo el liderazgo repercute en el empoderamiento estructural, influyendo consecuentemente en la satisfacción laboral, el compromiso y el aumento de la calidad de la asistencia prestada^(23)^.

Aunque la puntuación total del QUAPEEL no mostró correlación con el dominio “Oportunidad” del CET-II, una revisión integradora sobre el empoderamiento en el contexto de la urgencia y la emergencia señala que, para alcanzar la autonomía en el trabajo y mejores niveles de autoeficacia por las oportunidades, es fundamental que los enfermeros se basen en modelos positivos de liderazgo, como el resonante y el clínico^(13)^. Además, el aumento de las oportunidades de estos profesionales en la RUE puede percibirse en la Enfermería de Prácticas Avanzadas, lo que representa el crecimiento y el papel ampliado del enfermero en la APH brasileña^(24)^.

La Tabla 3 muestra cómo la “comunicación” en la autopercepción del enfermero es un componente significativo en el modelo de Liderazgo *Coaching,* ya que influye significativamente en su empoderamiento. La comunicación de los enfermeros líderes es crucial para construir buenas relaciones y empoderar a los miembros del equipo. Para ello, debe basarse en los pilares de la comunicación: un diálogo de apoyo, en lugar de impositivo; el intercambio de visiones y valores del líder y; la comunicación y el apoyo de prácticas basadas en la evidencia^(25)^.

Desde la lógica del liderado, dos trabajos^(4,9)^ que analizaron la percepción de los técnicos de enfermería sobre el ejercicio del Liderazgo *Coaching* por parte de sus coordinadores en el contexto APH móvil, la “comunicación” fue el segundo ámbito con mayor media, correlacionándose con el menor tiempo de formación^(4)^ y con la satisfacción en el trabajo^(9)^. La comunicación en el liderazgo coaching, según una revisión integradora reciente^(12)^, tiene un impacto significativo en el apoyo individual de los profesionales de enfermería liderados, lo que se refleja en el desarrollo de nuevas competencias de estos profesionales que les permiten ver nuevas formas de trabajar en equipo^(12)^.

Dar y recibir feedback del instrumento QUAPEEL solo se correlacionó con el poder informal, dominio del CET, lo que demuestra la relevancia de este instrumento en el proceso de empoderamiento. En este sentido, de los estudios coinciden en este resultado^(12,26)^. El primero analiza el liderazgo de los enfermeros de práctica avanzada y reveló que esta competencia, cuando se basa en el proceso de coaching, promueve el empoderamiento y la mejora del rendimiento del grupo, por lo que es evidente la necesidad de que los enfermeros valoren los mecanismos de feedback^(26)^.

Por su parte, el segundo trabajo, una revisión integradora^(12)^, destacó el feedback en el liderazgo *de coaching* como un facilitador para el empoderamiento de los enfermeros. Con ello, las organizaciones de salud y sus profesionales pueden tener un alto rendimiento y cumplir con los estándares y protocolos institucionales^(12)^. Es necesario que los enfermeros estén capacitados y dediquen el tiempo adecuado/apropiado al feedback, especialmente cuando se abordan aspectos como el error. Así, el líder debe ser capaz de ofrecer el apoyo necesario y promover una cultura de no castigo, además de analizar los fallos de forma sistemática^(25)^.

El poder formal, según el referente del empoderamiento estructural, comprende los aspectos relacionados con la posición jerárquica que un individuo ocupa dentro de una organización^(10)^. Este constructo del instrumento CET-II fue el único que se correlacionó con la dimensión del Liderazgo *Coaching* ”dar y ejercer influencia”, como se observa en la Tabla 3.

La influencia del enfermero es una habilidad que debe estimularse en los profesionales de las unidades de APH estudiadas, teniendo en cuenta los resultados de las correlaciones. Este hallazgo evidencia que la capacidad de inspirar y motivar a los equipos, junto con la competencia de comunicación eficaz y la toma de decisiones rápida, son fundamentales para garantizar la calidad de la atención y la seguridad del paciente en este contexto dinámico y desafiante que comprende la urgencia y la emergencia^(6,24)^.

Aunque la dimensión “Apoyar al equipo para alcanzar los resultados” presentó una menor correlación con los dominios del CET-II, un estudio reciente^(5)^ de la red de urgencias y emergencias de un municipio del interior de São Paulo señaló la necesidad de que el enfermero líder construya credibilidad para obtener el apoyo de todos los niveles de actuación, así como en todos los escenarios. Teniendo en cuenta que el SAMU y las UPA se inscriben en el contexto de la REU, para empoderarse, el enfermero necesita, en el ejercicio de su liderazgo, comprender las políticas internas y externas, analizar críticamente las demandas que surgen y reconocer el espacio y las oportunidades de liderazgo de terceros^(5)^.

La limitación de la investigación se relacionó principalmente con el número de profesionales que no se encontraban en sus turnos para ser invitados a participar en la investigación. Aun así, esta limitación no tuvo un gran impacto en los análisis estadísticos ni minimizó la relevancia y el carácter innovador de la investigación al presentar una investigación sobre el liderazgo de los enfermeros no solo en el contexto de la APH móvil, sino también en las Unidades de Atención de Urgencias Fijas, en la calidad de las UPA. Además, la realización del estudio permitió analizar la correlación de dos variables que tienen relevancia para la práctica profesional de los enfermeros de urgencias y emergencias.

Las contribuciones de la presente investigación demuestran la importancia de la práctica del liderazgo *coaching* en el empoderamiento de los enfermeros que trabajan en el contexto de la APH móvil y fija, destacando la correlación positiva entre la “comunicación” y todos los ámbitos del empoderamiento estructural. Este hallazgo tiene implicaciones directas en la práctica profesional, ya que sugiere que el desarrollo de habilidades comunicativas por parte de los enfermeros puede fortalecer su capacidad para influir y liderar al equipo de manera más eficaz, mejorando la organización y los resultados de la atención. Además, la relación obtenida entre “dar y recibir feedback” y el empoderamiento estructural refuerza la necesidad de prácticas de evaluación y apoyo continuo entre los profesionales.

## CONCLUSIÓN

Los hallazgos del presente artículo demuestran que el liderazgo de coaching ejercido por los enfermeros de la APH móvil y fija presenta una correlación positiva con el empoderamiento estructural. Este aspecto se observó por la correlación entre las puntuaciones totales de los instrumentos utilizados en la investigación, así como por las numerosas correlaciones entre los dominios del QUAPEEL y el CET-II. De este modo, estos hallazgos orientan las estrategias de capacitación centradas en elementos y competencias como la comunicación y el feedback, lo que repercute positivamente en la calidad de la atención, la satisfacción de los pacientes y el desarrollo profesional continuo de los enfermeros en la APH.

## Data Availability

Todo el conjunto de datos que apoya los resultados de este estudio fue publicado en el propio artículo.
